# Fibrinogen function indexes are potential biomarkers of diabetic peripheral neuropathy

**DOI:** 10.1186/s13098-021-00777-7

**Published:** 2022-01-18

**Authors:** Yong Zhuang, Xiahong Lin, Xiaoyu Chen, Xiaohong Wu, Jinying Zhang

**Affiliations:** 1 Department of Endocrinology, The Second Affiliated Hospital of Fujian Medical University, Quanzhou, 362000 China; 2 Department of Endocrinology, The Seventh Affiliated Hospital of Sun Yat-sen University, No. 950 Donghai Street, Fengze District, Quanzhou City, 518000 Fujian China; 3 Department of Neurology, The Second Affiliated Hospital of Fujian Medical University, Quanzhou, 362000 China

**Keywords:** Diabetic peripheral neuropathy, Fibrinogen, k value, Angle α, Diagnosis

## Abstract

**Background and objectives:**

Research suggests that diabetic peripheral neuropathy (DPN) is related to plasma fibrinogen (Fib) concentrations, although its correlation with Fib function has not been reported. Here, the k value and angle α, reflecting the plasma Fib function, were used to analyse its correlation with DPN, and their potential as biological indicators for diagnosing DPN was explored.

**Subjects and methods:**

This prospective observational clinical study enrolled 561 type 2 diabetes mellitus (T2DM) patients, who were divided into the diabetes with symptomatic neuropathy (161 cases), diabetes with asymptomatic neuropathy (132 cases) and diabetes with no neuropathy (268 cases) groups. Meanwhile, 160 healthy unrelated subjects were recruited as controls.

**Results:**

Fib levels increased slightly in diabetic subjects with neuropathy compared with those without. The angle α levels increased slightly in subjects with asymptomatic DPN compared with those with no neuropathy and increased greatly in subjects with symptomatic DPN compared with those without. The k value levels slightly decreased in subjects with asymptomatic DPN compared with those with no neuropathy and greatly decreased in subjects with symptomatic DPN compared with those without. The association of the k value and angle α with diabetic neuropathy was independent of the hyperglycaemic state and other potential confounders (odds ratio 0.080 [0.051–0.124], *P* < 0.001; odds ratio 1.131 [1.063–1.204], *P* < 0.001). The k value and angle α levels were closely correlated with neuropathy stage (*r*  = − 0.686, *P* < 0.000; *r * = 0.314, *P* < 0.001). The optimal cut-off point for k value levels to distinguish patients with diabetic neuropathy from those without was 1.8 min, with a sensitivity of 73.7% and a specificity of 83.2% (AUC = 0.873). The optimal cut-off point for angle α levels was 60°, with a sensitivity of 41.0% and a specificity of 95.6% (AUC = 0.669).

**Conclusions:**

The k value and angle α are closely associated with DPN. The levels of the k value and angle α may be helpful in the early diagnosis of DPN.

Diabetes mellitus (DM) is the fifth leading cause of death in the world, with an average global prevalence rate of 8.6% [1–3]. Diabetic peripheral neuropathy (DPN) is a common chronic complication of diabetes, with an incidence of 60 to 90%; nearly 50% of patients have no symptoms [4], and there is a high rate of disability.

The currently accepted and reliable methods for the diagnosis of DPN in the clinic are neuroelectrophysiological examination and small neurofibropathy examination [5, 6]. However, the examination is relatively time consuming, laborious, and costly, and it requires a professional doctor and a physician who can issue a report. Some scholars have tried to identify serological markers, such as neuron-specific enolase (NSE), Cys, TNF/TLR4 and transforming growth factor (TGF), for the diagnosis of DPN [7–9]. These markers have a certain diagnostic value, but none of them have become recognized diagnostic indicators.

At present, DPN is known to be associated with oxidative stress, metabolic abnormalities, and immune and inflammatory responses [10]. At the same time, studies have confirmed that neurological microcirculatory disorders are an important pathogenic mechanism [11]. In recent years, studies have found that haemodynamic disorders are involved in the pathogenesis of DPN [12]. Long-term hyperglycaemia in diabetic patients can activate glucose bypass metabolism through the polyol pathway, and glucose can be catalysed by aldose reductase to generate sorbitol, which can cause intracellular hyperosmosis and affect the balance of the intracellular environment, resulting in the degeneration and necrosis of nerve cells. Moreover, a high level of sorbitol can also promote excessive generation of oxygen free radicals in cells and damage the metabolism of nerve cells. In the long run, the excess of inflammatory mediators in the blood can damage the vascular endothelium and cause a state of inflammatory reaction in the body. Vascular endothelial damage and haemodynamic changes lead to hypercoagulability and microcirculation disorders. The inflammatory response, hypercoagulability and microcirculation disorders promote the occurrence and development of diabetic peripheral neuropathy. Plasma fibrinogen (Fib) is a kind of plasma glycoprotein synthesized and secreted by liver cells. Fib is the coagulation factor that has the highest concentration in plasma. Fib is not only the substrate of thrombin action but also the target of high-concentration fibrinolytic enzymes, and it participates in the coagulation process. Fib also reflects the inflammatory state of the body and plays an important role in the inflammatory response [13]. In addition, Fib binds to the receptor of β3 mucin and activates epidermal growth factor receptor on nerve cells, which inhibits the growth of nerve axons and is closely related to diabetic neuropathy. A previous study suggested that when coagulation and fibrinolysis function were abnormal in diabetic patients, the level of plasma Fib was significantly increased [14]. In addition, Fib is closely related to DPN [14–16]. In this regard, the k value and angle α as a function of Fib may act as novel emerging biomarkers of peripheral neuropathy in diabetes. However, to date, there has been no report on the relationship between Fib function and DPN. In addition, little data are available for Chinese individuals who face an increasing incidence of diabetes [17]. Therefore, we evaluated the relationship of the k value and angle α with diabetic neuropathy.

## Research design and method

### General information

A total of 561 subjects who met the 1999 World Health Organization (WHO) type 2 diabetes diagnostic criteria and were registered consecutively as outpatients or inpatients with our hospital along with 160 healthy control subjects registered with our hospital physical examination centre between August 2018 and March 2021 were randomly enrolled in the study. All volunteers signed informed consent. The study was approved by the hospital and university scientific and ethics committees. The exclusion criteria were age < 20 years or  > 75 years, severe liver or kidney damage, trauma, surgery, tumour, acute infection, pregnancy or lactation, diabetic ketosis, blood disease, long-term alcohol abuse, other nondiabetic causes (such as cerebral infarction, neck lumbar disease, severe infection, poisoning, malnutrition, etc.) that could cause neurological damage, and other diseases that may be confused with the clinical symptoms of DPN, such as vitamin deficiency, osteoarthritis, peripheral vascular disease, and trauma surgery. The inclusion criteria for healthy control subjects included no history of diabetes, fasting blood glucose < 5.6 mmol/L, and glycated haemoglobin < 5.6%.

### Method

#### Neurological symptoms and physical examination

Testing was performed on each participant by the same experienced physician according to standard procedure. All tests were conducted in a quiet laboratory. First, all patients had a complete history of neurological symptoms taken and were given a physical examination.

For somatic and cardioautonomic neuropathy, symptoms were documented, including numbness, paraesthesia, burning, deep aching, unsteadiness in walking, unexplained resting tachycardia and postural fainting [18].

The assessment by a professional medical staff member (Toronto clinical score) included 10 g nylon wire (pressure sense), tuning fork (vibration sense), temperature sense, acupuncture pain, and tendon reflex; positive findings on 2 of the 5 tests indicate abnormal signs of the nervous system [19, 20].

#### Nerve conduction velocity tests and clinical feature measurement

All patients were examined using an electromyography (EMG) instrument (Keypoint 9033A07, Denmark). All subjects were tested in a quiet environment. The distal latency (DML), motor conduction velocity (MCV) and motor nerve conduction amplitude (CMAP) of the bilateral median nerve, ulnar nerve, tibial nerve and common peroneal nerves were detected. The sensory conduction velocity (SCV) and sensory nerve action potential amplitude (SNAP) of the bilateral median nerve, ulnar nerve, superficial peroneal nerve and sural nerve were detected. At the same time, the bilateral tibial nerve H reflex and the ulnar nerve F wave latency were detected.

Body weight and upright height were measured on the same scales and wall-mounted stadiometer in light clothing without shoes before breakfast. Individual BMI was then calculated as weight (kg)/height (m)^2^. The right-arm blood pressure of each seated subject was obtained after 10 min of rest using a mercury sphygmomanometer. Retinal conditions were evaluated by ophthalmologists using a combination of clinical examination, stereoscopic retinal photographs, optical coherence tomography and fluorescein angiography.

All subjects stopped anticoagulant and antiplatelet drug use 2 weeks prior, and venous blood was collected in the morning from the antecubital vein after the subjects fasted for 10 to 12 h. The k value and angle α were assessed by a thromboelastography analyser (CFMS LBPU-8800; Lepu, Beijing). Fib was measured using a blood coagulation meter (FAC21A-UW; Ltd, Taiwan). Fasting plasma glucose, serum creatinine, blood lipids, and liver and kidney function were measured by an automatic biochemical analyser (Cobas 8000; Roche, Germany). HbA1c was measured using high-performance liquid chromatography (D10; Bio-Rad, Berkeley, CA). Serum vitamin B12 was measured using an automated assay (Maglumi 4000; China). The urinary albumin concentration was assessed using immunonephelometry (DCA2000; Bayer, Leverkusen, North Rhine–Westphalia, Germany). The urinary creatinine concentration was quantified using the alkaline picrate method. The individual urinary albumin-creatinine ratio (UACR) was then calculated as albumin (mg)/creatinine (g). Endogenous creatine clearance (Ccr) was calculated to estimate the glomerular filtration rate according to the Cockcroft equation: Ccr = {[140 – age (years) × body weight (kg)]/[0.818 × serum creatinine (Scr, µmol/L)]} for males and × 0.85 for females.

#### Diagnosis and stages of polyneuropathy

Diabetic neuropathy was diagnosed according to the American Diabetes Association recommendation [21]. Polyneuropathy was further staged into three groups. Group A (symptomatic neuropathy) was defined as the diabetes with symptomatic neuropathy group. Group B (asymptomatic neuropathy) was defined as the diabetes with asymptomatic neuropathy group. Group C (no neuropathy) was defined as the diabetes without neuropathy group. Group D (no neuropathy) was a healthy control group.

### Statistical analysis

We used SPSS version 19 for statistical analysis. The data are expressed as the mean (SD) for normally distributed data. The chi-square test was used to compare the count data. Multiple comparisons among groups were assessed using one-way analysis and comparisons between two groups (LSD method) for variables. A t test was used for comparison between the two groups. k value and angle α were later added to a logistic regression model, controlling for possible confounders. The relation of the k value and angle α levels to the stages of neuropathy was calculated using Spearman’s correlation analysis. Receiver operating characteristic (ROC) analysis was conducted with MedCalc Software version 15.2 to assess the accuracy of serum k values and angle α levels in distinguishing between patients with and without diabetic neuropathy. The optimal cut-off point was identified by calculating the area under the curve (AUC). *P* < 0.05 was considered indicative of statistical significance.

## Results

**Fig. 1:**
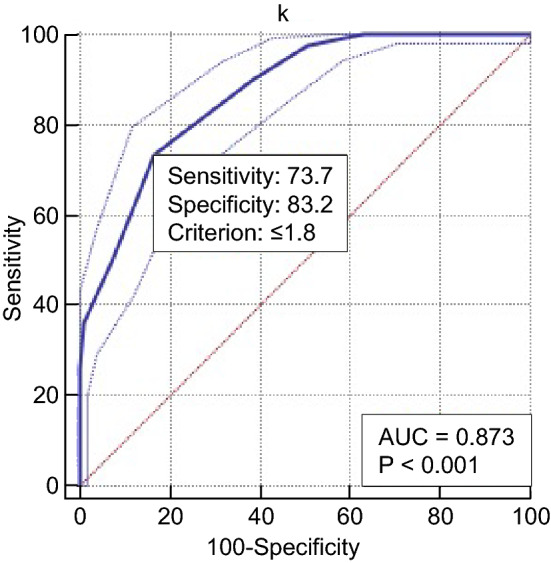
ROC curve of k value

**Fig. 2:**
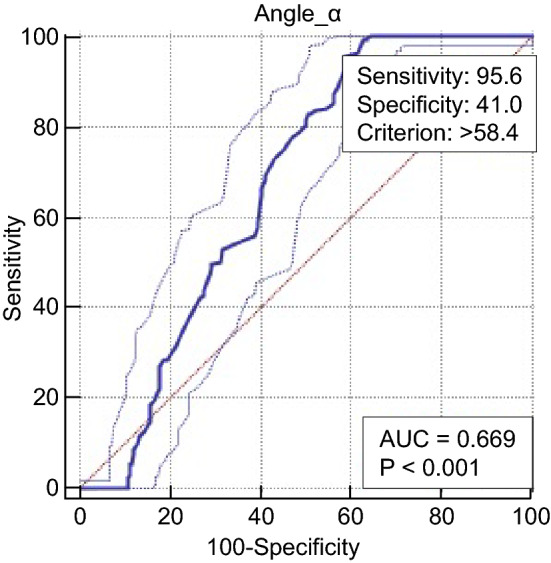
ROC curve of Angle α

The study was completed by 561 subjects, including 160 healthy control subjects, 293 diabetic subjects with neuropathy and 268 diabetic subjects without neuropathy (Table 1). Among the four groups of subjects, there were no differences between any two groups in the following variables: sex ratio, age, blood pressure, BMI, blood lipids (total cholesterol, low-density lipoprotein (LDL) cholesterol, high-density lipoprotein (HDL) cholesterol), liver and kidney function (alanine transaminase (ALT), aspartate transaminase (AST), urinary albumin-to-creatinine ratio (UACR) and Ccr), vitamin B12 and platelet count (PLT). The incidence of diabetic retinopathy was higher in the diabetic neuropathy group than in other groups. A comparison of diabetes among the groups revealed that that diabetic neuropathy group (group A) had the longest course of all groups (Table 1). Diabetic subjects with symptomatic neuropathy showed increased levels of Fib compared with patients without neuropathy and those with asymptomatic neuropathy. There was no significant decrease in the k value relative to the normal control group (group D) in the diabetic group without peripheral neuropathy (group C) (*P* = 0.191). The k value was slightly reduced in the subclinical DPN group (group B) (*P* < 0.001) and further decreased in the confirmed DPN group (group A) (*P* < 0.001). Compared with the normal control group (group D), the angle α levels were not significantly increased in the diabetic group without DPN (group C) (*P* = 0.404), but there was a significant increase in the subclinical group (group B) and the confirmed DPN group (group A) (*P* < 0.001). Moreover, the confirmed DPN group (group A) had significantly higher angle α levels than the subclinical DPN group (group B) (P = 0.002) (Table 2). The k value and angle α levels were further assessed in relation to neuropathy in a multivariate model (Table 3), controlling for retinopathy and other covariables that may potentially influence the k value and angle α levels or neuropathy, including the disease course, age, HbA1c, estimated glomerular filtration rate (eGFR), UACR, vitamin B12 and Fib. After adjustment, the k value and angle α were still independently associated with diabetic neuropathy (odds ratio 0.080 [0.051 ~ 0.124], *P* < 0.001; odds ratio 1.131 [1.063 ~ 1.204], *P* < 0.001, respectively). Correspondingly, k value levels were negatively correlated with diabetic peripheral neuropathy (*r* = − 0.656, *P* < 0.001), and angle α levels were positively correlated with diabetic peripheral neuropathy (*r* = 0.314, *P* < 0.001). Fib levels were also correlated with diabetic peripheral neuropathy (*r* = 0.252, *P* < 0.001) (Table 3). The k value and angle α levels were shown to distinguish between patients with and without diabetic neuropathy (Table 4). The optimal cut-off points were 1.8 min for the k value, with a sensitivity of 73.7%, a specificity of 83.2%, and the highest AUC equal to 0.873 (*P* < 0.001), and 58.4° for the angle α with a sensitivity of 95.6%, a specificity of 41.0%, and the highest AUC equal to 0.669 (*P* < 0.001) (Figs. 1, 2).

**Table 1:** Comparison of clinical features between different groups

Group	A	B	C	D	*P* value
Case (male/female)	82/79	70/62	140/128	84/76	0.145
Age (years)	50.7 ± 7.6	51.3 ± 8.0	51.2 ± 8.5	51.1 ± 7.0	0.839
Disease course (years)	8.5 ± 3.6	7.8 ± 2.9	6.6 ± 3.4	–	< 0.001
SBP (mmHg)	122 ± 9	122 ± 9	123 ± 9	121 ± 10	0.476
DBP (mmHg)	70 ± 7	70 ± 6	69 ± 7	68 ± 6	0.206
BMI (kg/m^2^)	23.9 ± 1.7	24.0 ± 1.7	23.9 ± 1.7	23.9 ± 1.9	0.928
FPG (mmol/L)	8.3 ± 1.7	8.2 ± 1.7	8.4 ± 1.4	4.8 ± 0.5	< 0.001
HbA1c (%)	8.2 ± 1.3	8.2 ± 1.4	8.0 ± 1.5	4.8 ± 0.5	< 0.001
TC (mmol/L)	4.9 ± 0.8	4.9 ± 0.6	4.8 ± 0.7	4.8 ± 0.9	0.967
LDL-C (mmol/L)	2.7 ± 0.9	2.7 ± 0.9	2.8 ± 0.7	2.6 ± 0.7	0.391
HDL-C (mmol/L)	1.4 ± 0.4	1.3 ± 0.4	1.4 ± 0.4	1.4 ± 0.4	0.397
ALT (IU/L)	23 ± 3	24 ± 2	24 ± 3	23 ± 4	0.233
AST (IU/L)	22 ± 4	22 ± 3	22 ± 3	22 ± 3	0.411
Vit B12 (pmol/L)	522 ± 185	521 ± 201	542 ± 204	525 ± 187	0.607
PLT (× 10^9^/L)	233 ± 70	237 ± 66	238 ± 62	246 ± 66	0.329
DR (%)	23.6	21.2	11.2	–	0.002
UACR (mg/g)	21.3 ± 3.5	22.0 ± 2.9	21.4 ± 3.2	–	0.101
eGFR [ml/(min 1.73m^2^)]	98.7 ± 26.7	104.3 ± 29.0	103.1 ± 26.7	100.4 ± 33.6	0.291

**Table 2:** Comparison of k value, angle α and Fib between groups

Group	Fib (g/L)	k value (min)	Angle α (°)
A	3.54 ± 0.45	1.6 ± 0.2	61.9 ± 1.5
B	3.07 ± 0.56	1.8 ± 0.1	60.7 ± 1.7
C	3.11 ± 0.60	2.1 ± 0.3	59.3 ± 4.5
D	3.05 ± 0.60	2.1 ± 0.3	59.6 ± 3.4
*P* value^1^	< 0.001	< 0.001	0.002
*P* value^2^	< 0.001	< 0.001	< 0.001
*P* value^3^	< 0.001	< 0.001	< 0.001
*P* value^4^	< 0.001	< 0.001	< 0.001
*P* value^5^	0.482	< 0.001	0.005
*P* value^6^	0.322	0.191	0.404

(^1^*P*, diabetes with symptomatic neuropathy vs. diabetes with asymptomatic neuropathy. ^2^*P*, diabetes with symptomatic neuropathy vs. diabetes without neuropathy. ^3^*P*, diabetes with ymptomatic neuropathy vs. healthy control. ^4^*P*, diabetes with asymptomatic neuropathy vs. diabetes without neuropathy. ^5^*P,* diabetes with asymptomatic neuropathy vs. healthy control. ^6^*P,* diabetes without neuropathy vs. healthy control.)

**Table 3:** Multiple regression analysis of the relation of k value and angle α to neuropathy

Covariables	*OR*	*95% CI*	*P* value
Disease course	1.106	1.025–1.194	0.010
Age	0.985	0.959–1.011	0.254
HbA1c	1.100	0.944–1.282	0.220
DR (%)	1.219	0.617–2.409	0.569
eGFR	0.999	0.991–1.007	0.754
UACR	1.055	0.987–1.128	0.116
Vit B12	0.999	0.9984–1.001	0.319
Fib	1.543	1.069–2.227	0.021
Angle α	1.131	1.063–1.204	< 0.001
k value	0.080	0.051–0.124	< 0.001

**Table 4:** Correlation analysis between diabetic peripheral neuropathy and plasma k value and angle α (spearman correlation analysis)

Item	Angle α	k value	Fib
DPN			
*r*	0.314	− 0.656	0.252
*P* value	< 0.001	< 0.001	< 0.001

## Discussion

The results of this study demonstrated that the k value and angle α are potential biomarkers of DPN. In this study, patients with DPN had lower k values than diabetic patients without peripheral neuropathy, and the levels of angle α in patients with DPN were significantly higher than those in diabetic patients without peripheral neuropathy. More importantly, the k value and angle α were changed in the early stage of diabetic peripheral neuropathy (subclinical diabetic peripheral neuropathy).

DPN refers to clinical and/or electrophysiological evidence of peripheral neuropathy in patients with a confirmed diagnosis of diabetes, excluding other diseases. The incidence of DPN can be as high as 60 to 90%, and the incidence rate reported in China is as high as 85%. At present, the pathogenesis of DPN remains unclear. Many studies suggest that DPN is associated with abnormal metabolic pathways, microvascular disease, nerve growth factor, autoimmunity, inflammation and oxidative stress. Among them, metabolic pathway abnormalities and microvascular changes are considered to play an important role in the occurrence and development of DPN [22, 23]. A large number of studies have shown that there are abnormalities in blood coagulation and fibrinolysis in diabetic patients, and a hypercoagulable state is present [14, 24]. Furthermore, in patients with diabetes, insulin resistance is present. When blood glucose control is poor, high insulin and high blood sugar levels increase the level of plasminogen activator inhibitors, and at the same time, the secretion of plasminogen activator inhibitors from vascular endothelial cells increases the hypercoagulable state, leading to the occurrence and development of diabetic peripheral neuropathy [25]. Fib is enhanced when there is hypercoagulability and hyperfibrinolysis in the blood. Erem C [26] found that patients with type 2 diabetes were hypercoagulable and that Fib was significantly higher in patients with type 2 diabetes mellitus with peripheral neuropathy than in those without neuropathy, which is consistent with the results of this study. However, the traditional Fib assay can only measure the amount of Fib in blood in vitro but does not assess its function. The k value and angle α mainly reflect Fib function, and the high Fib functional state is characterized by a shortened k value and an increased angle α. This study found that the k value was lower in diabetic patients with peripheral neuropathy than in normal subjects and those without peripheral neurological complications. This suggests that the formation of Fib is short and that the speed is increased when diabetic peripheral neuropathy occurs. The angle α level in diabetic patients with peripheral neuropathy is higher than that in normal subjects and those with uncomplicated diabetes, suggesting that Fib function is enhanced when there are complications of peripheral neuropathy. Moreover, in this study, the k value and the angle α levels were closely related to the degree of diabetic neuropathy; the k value levels were reduced in subclinical diabetic peripheral neuropathy and further decreased as the degree of diabetic peripheral neuropathy increased. The angle α levels increased as the degree of neuropathy increased. This relationship was independent of covariables.

Diabetic peripheral neuropathy has a high incidence, and its clinical symptoms are also diverse. Due to the low degree of neurological damage in the early stage of DPN, typical symptoms and signs are not evident, and no symptoms may be present. This is easily overlooked, and once the disease progresses, there may be adverse outcomes such as diabetic foot [4]. Therefore, the diagnosis of diabetic peripheral neuropathy, especially the early diagnosis, is crucial.

At present, the most commonly used methods for the diagnosis of DPN are neuromuscular electromyography (neural conduction measurement), nerve biopsy, the Toronto clinical score and the Michigan neuropathy screening table, all of which have limitations and low clinical support. According to different screening methods, the prevalence of DPN was 2.4–74.8% [27]. In contrast, serological diagnostic markers are time-saving, labour-saving, and inexpensive, have high clinical support and do not require professional physicians to perform the assays. Currently, however, there are few studies on serological markers for the early diagnosis of DPN (such as cystatin C and NSE) [6, 7]); thus far, they have not become globally recognized indicators in the diagnosis of DPN [28]. In this study, we further confirmed that Fib was elevated in diabetic peripheral neuropathy. In addition, we found that the k value and angle α were closely related to DPN and that there was a significant change in subclinical DPN. The results suggested that subclinical DPN had a smaller k value and an increased angle α, which can provide a basis for the earlier diagnosis of DPN. At the same time, this study concluded that the optimal cut-off points for the k value and angle α levels to distinguish patients with diabetic neuropathy from those without diabetic neuropathy were 1.8 min (≤ 1.8 min) and 58.4° (> 58.4°), respectively. Neither of these two intercept points exceeds the reference range of k value and angle α (k: 1–3 min; angle α: 53–72°); thus, if the lower limit of the reference value range of the k value is used as the reference boundary value to distinguish the presence or absence of diabetic peripheral neuropathy, it will be too low in patients with diabetic peripheral neuropathy; likewise, if the upper limit of the reference range of the angle α is taken as the boundary value, it will be too high in DPN patients. This is not conducive to the early diagnosis of diabetic peripheral neuropathy.

## Conclusions

This study further confirmed that abnormal blood coagulation may lead to the occurrence and development of diabetic peripheral neuropathy. The k value, angle α and Fib serve as indicators of coagulation function and may serve as potential blood biomarkers for the diagnosis and treatment of diabetic peripheral neuropathy. Importantly, the k value and angle α may be helpful for the early diagnosis of diabetic peripheral neuropathy. Clinicians can monitor the k value, angle α and Fib to detect diabetic peripheral neuropathy in time. When the monitoring indicators are abnormal, adding drugs to improve blood viscosity on the basis of diabetes treatment may have a certain role in preventing and delaying the occurrence and progression of diabetic neuropathy, benefitting the patients. However, there are still some limitations to this study. The sample size of this study was not large, which may have had an impact on the research. Further comprehensive research with large sample sizes is needed.

## Data Availability

The datasets used or analysed during the current study are available from the corresponding author on reasonable request.
